# Oral administration of bromelain and acetylcysteine in pseudomyxoma peritonei (PMP) caused by low-grade appendiceal mucinous neoplasm (LAMN): a case report

**DOI:** 10.1186/s13256-023-04024-7

**Published:** 2023-06-29

**Authors:** Dominik Geisel, Ute Langen, Thomas Rüdiger

**Affiliations:** Department of Radiology, Charité, Universitätsmedizin Berlin, Campus Virchow-Klinikum, Klinik Für Radiologie, Augustenburger Platz 1, 13353 Berlin, Germany

**Keywords:** Acetylcysteine, Bromelain, PMP, LAMN

## Abstract

**Background:**

Pseudomyxoma Peritonei (PMP) is a severe neoplastic clinical syndrome characterised by secretion of mucin from tumors often originating in the appendix. The standard treatment includes cytoreductive surgery (CRS) combined with heated intraperitoneal chemotherapy (HIPEC). A new perspective in PMP treatment aims at the mucins themselves as a therapeutic target.

**Case presentation:**

Here we report the first case of PMP with peritoneal dissemination of mucinous implants caused by low-grade appendiceal mucinous neoplasm (LAMN) in a 58-year-old white male exclusively treated by appendectomy and oral administration of bromelain and acetylcysteine in the context of a medical self-experimentation (by co-author T.R.). Observation so far covers a period of 48 months including regular magnetic resonance imaging (MRI) with stable findings.

**Conclusions:**

Oral administration of bromelain and acetylcysteine can be used in the treatment of PMP caused by LAMN without relevant clinical side effects.

## Introduction and background

The term PMP describes the peritoneal dissemination of mucus, gelatinous ascites, and mucinous implants. When left untreated, PMP leads to bowel obstruction, anorexia, and death due to large amounts of mucinous ascites.

Mucinous neoplasms of the appendix such as low-grade appendiceal mucinous neoplasm (LAMN) are the primary cause of PMP. If LAMN leads to rupture of the appendix, it may be followed by PMP with diffuse peritoneal dissemination [1].

The current standard treatment consists of two components, firstly so-called cytoreductive surgery (CRS) and secondly heated intraperitoneal chemotherapy (HIPEC). CRS aims to remove all visible intraperitoneal tumor metastases, that is complete macroscopic cytoreduction. Therefore, an aggressive surgical procedure is performed, including not only extensive peritonectomy, but also omentectomy, splenectomy and, depending on tumor extension, various organ removals such as hemicolectomy or gastrectomy. Surgery times of around 12 h are therefore not uncommon [2].

The outcomes after CRS and HIPEC were compared with those after CRS alone in a retrospective cohort study based on the Peritoneal Surface Oncology International Registry despite the lack of randomized data [3]. The study included 1924 patients with PMP due to an appendiceal neoplasm. The weighted 5-year overall survival for CRS alone was 46.2%. CRS combined with HIPEC was associated with better-weighted 5-year overall survival (57.8%). Based on the whole study population (*n* = 1924), incidences of 4.2% for 90-day mortality and 32.0% for severe morbidity were reported.

After CRS and HIPEC, recurrence of PMP was examined in an observational study based on a prospective multicenter national database [4]. After exclusion of patients who underwent either incomplete cytoreduction or no HIPEC (*n* = 394) and patients with missing data (*n* = 69), a total of 948 patients could be evaluated in this study. It was reported that 229 patients (24.4%) developed a recurrence and that 196 (85.6%) of those occurred before the 5-year follow up, and that the mean time to recurrence was nearly 2.5 years.

In recent years, research into secreted mucins in PMP has become the focus of attention [5–9]. In particular, research into drugs that specifically dissolve the mucus in PMP has been intensified. Basic preclinical research results of mucolysis in PMP were published 2016 in a monograph by Amini *et al. *[10]. *In vitro* and *in vivo* formulation studies of bromelain and acetylcysteine were shown to exhibit a strong synergistic mucolytic potency.

Bromelain is extracted from the stems of the pineapple plant (*Ananas comosus*). It is a mixture of enzymes with proteolytic and non-proteolytic properties. Bromelain is an orally-given drug licensed in Germany for edema-reduction and edema-prevention after surgery or injuries. It has several additional pharmacological systemic effects, for example inhibition of platelet aggregation and stimulation of fibrinolysis (reviewed by Mauer 2001 [11]). Therefore it should not be used in patients with blood clotting disorders, nor in patients taking anticoagulants or thrombocyte-aggregation inhibitors.

Acetylcysteine is a well-known and widely used orally-given mucolytic drug, used especially in lung diseases such as bronchitis and cystic fibrosis.

Both drugs received an orphan medical product designation for treatment of PMP in 2018 by the European Commission, based on the vote of the Committee for Orphan Medicinal Products of the European Medicines Agency. Starting in 2018, a phase I (first in man) study was performed in patients who were not suitable for CRS and HIPEC or in whom these therapies had failed [12]. In that study an intraperitoneal injection of these drugs was used.

## Case presentation

**Fig. 1:**
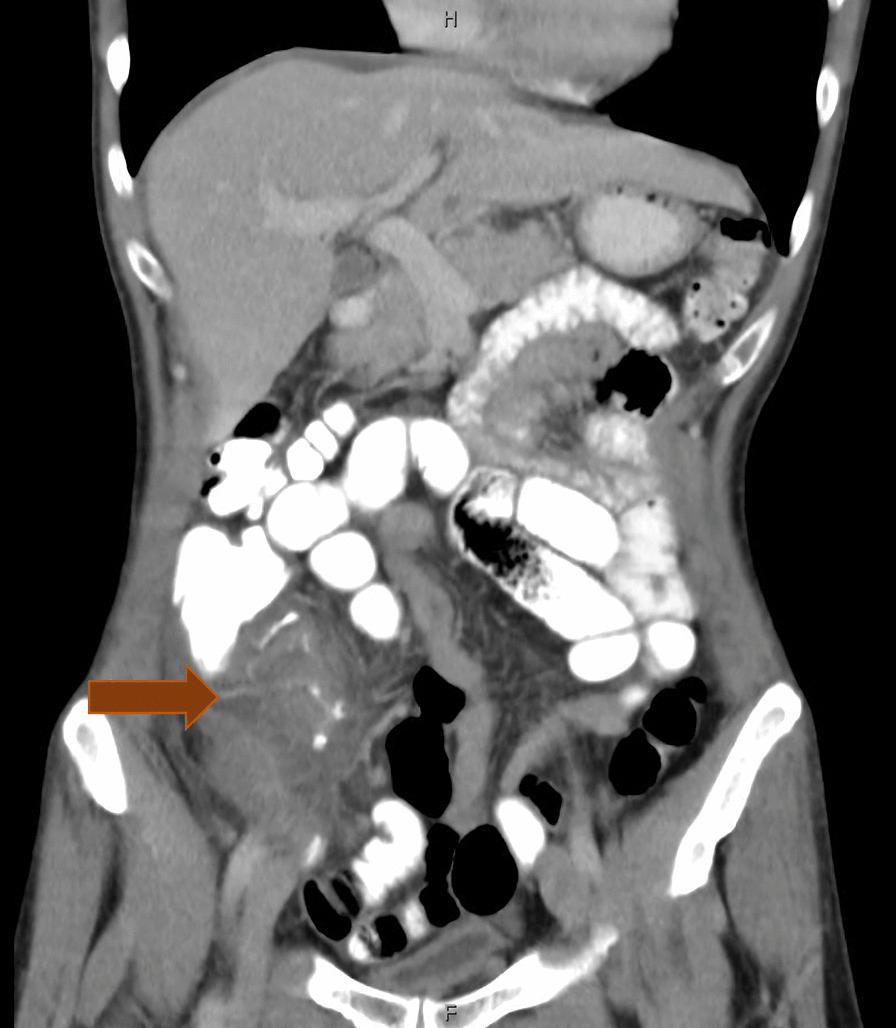
Contrast-enhanced coronar CT scan of the abdomen and pelvis shows mucinous mass of the coecum and appendix (red arrow)

A 58-year-old white male without known concomitant diseases underwent emergency laparoscopic appendectomy for suspected acute appendicitis in March 2018. Intraoperatively, only a mucocele of the appendix was diagnosed. Clinical findings worsened on postoperative day 8, so that hospitalization and intravenous antibiotics were administered under the suspected diagnosis of peritoneal phlegmon (Fig. 1). After clinical improvement, MRI showed mucinous implants in all four quadrants of the abdomen. Final histology revealed a LAMN with acellular mucus on the surface of the appendix (pT4a) and a proliferation rate of 50% of Ki-67. The tumor board’s recommendation for CRS and HIPEC was not followed by the patient because he accepted the need for treatment on the one hand, but on the other hand, knowing the current literature at that time, wanted a less invasive treatment option. The search for this less invasive treatment method led to the start of an oral mucolysis treatment 4 months after primary diagnosis. This time was required for literature search and evaluation by the co-authors.

Due to subjective freedom from symptoms and generally unremarkable blood values (for example blood count, liver enzymes, blood coagulation and so on), mucolytic therapy was started with oral administration of Bromelain 3 × 800 FIP (Federation International Pharmaceutique) units and 3 × 600 mg Acetylcysteine per day.

**Fig. 2:**
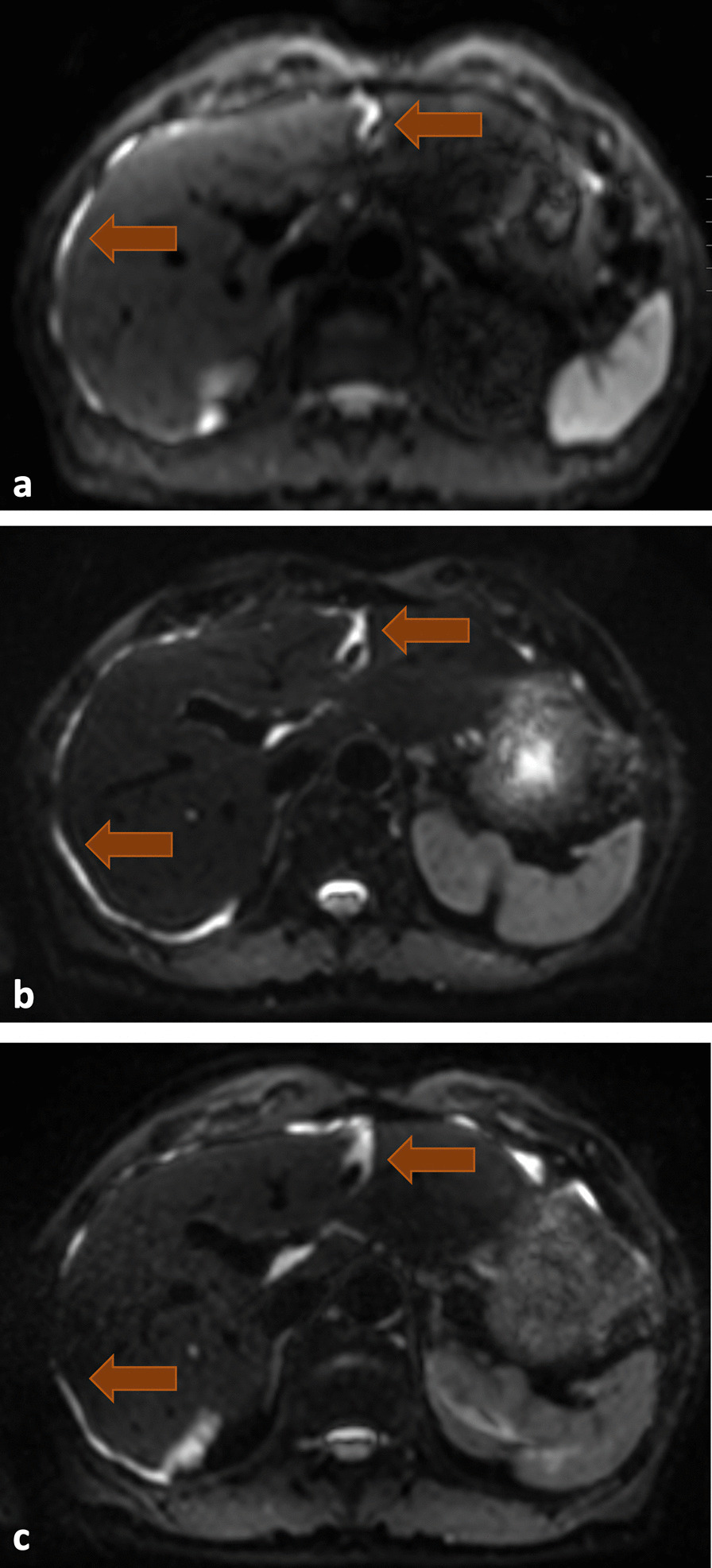
MRI scan with diffusion-weighted imaging (DWI) sequence shows mucinous implants as bright spots around the liver and in the falciforme ligament (red arrows). Subfigure **a** shows initial findings, **b** 1 year afterwards and c shows the stable findings 4 years after initial diagnosis

Based on good clinical tolerability, the bromelain dose was increased at the beginning of 2019 to 5 × 800 FIP units per day. Since the beginning of 2021, however, the drug had to be replaced by a preparation with 500 FIP units per tablet due to supply difficulties, resulting in a dosage of 5 times 1000 FIP units per day. There was no concomitant medication with exception of vitamin D substitution. The mucolytic therapy was well tolerated without any side-effects, including blood coagulation and fibrinolysis. Follow-up by MRI was initially performed every six months, and annually since 2020. Figure 2 shows the course of the perihepatic mucinous manifestations.

No serologic tumor marker determinations were performed.

The clinical course was inconspicuous during the entire observation period, that is the patient was completely free of symptoms and able to perform according to his age, including his professional medical activity.

## Discussion

This case report describes the clinical course of a LAMN patient who at primary diagnosis already had PMP with mucinous implants in all 4 quadrants of the abdomen, and in whom standard treatment consisting of CRS and HIPEC was abandoned in favor of oral mucolysis although Ki-67 was very high at 50%. Arjona-Sanchez *et al.* 2021 [13] described Ki-67 levels above 15% as a negative prognostic factor in high-grade PMP patients with reduced 5-year overall survival and reduced recurrence-free survival. In contrast to this, Ward *et al.* 2021 [14] state that a median Ki-67 score of 15% and a hotspot Ki-67 score of 50% were not predictive for progression-free survival after CRS and HIPEC. The possibility of “expectant observation” has been described only for patients with limited extent of disease, defined as 1 or 2 quadrants with small-volume disease [15]. In contrast, “diffuse” extent was defined as disease burden (implants) in more than 2 quadrants. Therefore, further treatment after appendectomy was necessary, but the patient—himself a physician with experience in oncologic surgery—demanded as few quality-of-life restrictions as possible through the therapy. Currently, the results of Kusamura *et al.* 2021 on the efficacy of CRS and HIPEC ([3], described in the introduction) were commented on by Ke *et al.* 2021 [16] as follows: “In a multicentre database of 1411 CRS + HIPEC procedures performed over two decades, it was reported that the rate of early recurrence in the first 5 years post-procedure was 25%. However, on closer inspection, the true figure is much higher, as it was reported that of the 262 patients followed up for over 10 years, 192 patients (75%) experienced a recurrence in the first 5 years, and only 33 patients (13%) had no recurrence after 10 years [4]”. In addition, they refer to the fact that repeat surgeries are more surgically complex, confer increased morbidity, and can usually be performed only 2 or 3 times before patients are inoperable. They also describe effects on various blood parameters (CRP, albumin, white cell count, creatinine) and the concentrations of bromelain in the blood after intraperitoneal administration of bromelain and acetylcysteine. Conversely, this also indicates drug levels in peritoneal fluid after oral administration of bromelain and acetylcysteine.

Dosages used in the case presented here were theoretically derived from preclinical experiments with the so-called PMP model, in which the decrease in Ki-67 positive cells was evaluated in addition to other parameters such as tumor weight, and compared with the pharmacokinetic and pharmacodynamic properties of the active substances [10]. Both agents are approved as drugs for oral treatment in Germany, albeit for different indications and at different doses. For the treatment of PMP, especially in recurrence, different treatment approaches have been reported, for example a combination of celecoxib and Myrtol [17], trifluridine/tipiracil (TAS-102) and bevacizumab [18]. A review of novel perspectives in PMP treatment can be found in Sommariva *et al.* 2021 [9], which also includes a section dedicated to “Mucin as a therapeutic target”. In addition to the first results of a phase I first in man study on intraperitoneal application of bromelain and acetylcysteine [12], two cases of intrathoracic application of these agents have already been reported [19]. Mucolytic therapy of PMP requires further evaluation to clarify its place in the therapy of PMP.

## Conclusion

Oral administration of bromelain and acetylcysteine can be used in the treatment of PMP caused by LAMN. Relevant side effects were not observed during the observation period of more than 4 years. Further studies on mucolysis with bromelain and acetylcysteine with regard to mode of application, best possible selection of patients, appropriate time of therapy initiation (preoperative and/or postoperative), optimal dosage, measurement of therapeutic success, and side effect profile are needed.

## Data Availability

All data generated or analysed during this study are included in this article. Further enquiries can be directed to the corresponding author.
